# Alleviating Heat Stress in Fattening Pigs: Low-Intensity Showers in Critical Hours Alter Body External Temperature, Feeding Pattern, Carcass Composition, and Meat Quality Characteristics

**DOI:** 10.3390/ani14111661

**Published:** 2024-06-01

**Authors:** José Segura, Luis Calvo, Rosa Escudero, Ana Isabel Rodríguez, Álvaro Olivares, Beatriz Jiménez-Gómez, Clemente José López-Bote

**Affiliations:** 1 Food Technology Department, Faculty of Veterinary Medicine, Complutense University of Madrid, Av. Puerta de Hierro s/n, 28040 Madrid, Spain; luiscalvo@incarlopsa.es (L.C.); mariab09@ucm.es (B.J.-G.); 2 Incarlopsa, N-400, Km 95.4, 16400 Tarancón, Spain; airodriguez@incarlopsa.es; 3 Animal Production Department, Faculty of Veterinary Medicine, Complutense University of Madrid, Av. Puerta de Hierro s/n, 28040 Madrid, Spain; rmescude@ucm.es (R.E.); alolivares@ucm.es (Á.O.); clemente@ucm.es (C.J.L.-B.)

**Keywords:** swine, heat stress, low-intensity shower, body temperature, feeding pattern, pork, desaturase

## Abstract

**Simple Summary:**

Pigs lack functional sweat glands, hence being very susceptible to heat stress. The optimum temperature in which pigs would thrive is around 20 °C, depending on age and weight. Pigs could achieve higher heat dissemination, e.g., by increasing body contact with the cooler ground and panting, but also through an undesirable reduction in feed intake. In addition, under conditions of severe heat stress, animals may become more susceptible even to immune challenges due to damage to the structure of the intestinal wall. Thus, heat stress can affect the proper functioning of metabolism, which, in addition to having implications on animal welfare, also affects the quality of meat and meat products. Cooling systems are not yet commonly seen on farms. Sprinkler systems are the most used cooling system, followed by water pads and fogging systems, for finishing pigs. As our climate continues to warm, monitoring daily feed intake and water consumption levels, along with the implementation of cooling systems, must become tools to minimize the adverse effects of hot weather.

**Abstract:**

Heat stress is a significant environmental problem that has a detrimental impact on animal welfare and production efficiency in swine farms. The current study was conducted to assess the effect of low-intensity showers, provided during critical high-temperature hours daily, on body external temperature, feeding pattern, and carcass and meat quality characteristics in fattening pigs. A total of 400 animals (200 barrows and 200 gilts) were randomly allotted in 40 pens. A shower nozzle was installed over 20 pens (half barrows and half gilts) where pigs received a low-intensity shower for 2 min in 30 min intervals from 12 to 19 h (SHO group). Another group without showers was also considered (CON). Feeder occupancy measurement, thermographic measures, and carcass and meat quality parameters were studied. In the periods with higher environmental temperatures, SHO animals showed an increase in the feeder occupancy rate compared to the CON group. A decrease in temperature was observed after the shower, regardless of the anatomical location (*p* < 0.005). The treatment with showers led to higher values than in the CON group of 4.72%, 3.87%, 11.8%, and 15.1% for hot carcass weight, lean meat yield, and fat thickness in *Longissimus Dorsi* (LD) and *Gluteus Medius* muscles, respectively (*p* < 0.01). Pork from CON showed a 14.9% higher value of drip loss, and 18.9% higher malondialdehyde concentration than SHO (*p* < 0.01); meanwhile, intramuscular fat content was 22.8% higher in SHO than in CON (*p* < 0.01). On the other hand, the CON group exhibited higher L* (2.13%) and lower a* and b* values (15.8% and 8.97%) compared to the SHO group. However, the pH_20h_ of the CON group was significantly lower than that of the SHO group (*p* < 0.001), indicating a softer pH decrease. Related to fatty acids in subcutaneous outer and inner layers and intramuscular fat, the CON group showed higher ΣSFA and lower ΣMUFA and Δ_9_-desaturase indexes than SHO (*p* < 0.05). In conclusion, the amelioration of heat stress through showers at critical times should be considered an interesting tool that improves both carcass and meat quality, as well as animal welfare.

## 1. Introduction

The detrimental effects of heat stress on animal welfare and production will become more of an issue if the Earth’s climate continues to warm as predicted.

Pig heat stress (HS) results in compromised welfare, with negative impact on behavior and health, thus decreasing productivity by reducing growth rate [1,2]. This alters the flow of animals from the farm to the industry (affecting industry decision making) and decreases carcass and meat quality characteristics.

Pigs are poorly adapted to HS due to predominant subcutaneous fat accumulation which impairs body core heat dissipation, and a limited number of functional sweat glands, which makes it difficult for them to regulate their body temperature through evaporation [3]. Therefore, a pig must resort to panting to dissipate heat, with concomitant metabolic alkalosis consequences and decrease in feed intake (FI), which reduces digestive and metabolic heat production. The amount of ingested feed and analysis of feeding behavior have been shown to be related to body core temperature [4,5]. The highest critical temperature that triggers adaptative short-term mechanisms in fattening pigs has been proposed to be in the range 25–26 °C [3]. However, at later fattening stages (>70 kg), the effect of HS on FI is more marked both in terms of higher critical temperature and the adaptative response on lowering FI per increased degree [5].

In a short HS challenge, when temperature returns to thermoneutrality, pigs increase intake rapidly to reach similar levels to that of unchallenged pigs. On the other hand, under ad libitum feeding of fattening pigs, most intake takes place during the day/light hours, and feeding pattern is characterized by two peaks of activity, one in the early morning and a larger one in the late afternoon [6]. The size of the peaks and the sinking of the intermediate valley become more evident as the fattening period progresses [7,8] and temperatures rise at midday. High demand for the feeder during peak hours leads to small displacements and shorter feeding visits [9]. Therefore, an HS situation during most light hours leads to high demand for feeder occupation during the coolest light hours and a moderate increase in night hour intake, which altogether does not allow fully compensation of daily intake [10].

In production settings, lower gain-to-feed ratio (G/F), average daily gain (ADG), and average daily feed intake (ADFI) values of growing and finishing pigs in higher ambient temperatures have been widely reported [11,12]. Rauw et al. [13] also pointed out that a yield increase of different primal cuts could be directly related to the period during production that HS occurred (growing, fattening, or finishing). A great impact of HS on muscle metabolism and its effect on meat quality characteristics have also been observed that may be attributed not only to lower FI, but to alkalosis and production of reactive oxygen species [14].

During the last decade, several strategies have been studied to alleviate the effects of HS on pig-feeding behavior [15]. Gourdine et al. [3], on the basis that genetic selection of genotypes tolerant to HS is a promising long-term option, reviewed the latest knowledge on the genetics of thermoregulation in pigs. In the case of dietary modifications, Dos Santos et al. [4] compared the conventional two-phase system to an individualized daily-precision feeding system with tailored dietary amino acid inclusion levels. Additionally, Fraga et al. [16] studied responses to sequential feeding with high-fat/low-crude protein diets, and Bin-Jumah et al. [17] reviewed the use of chromium to relieve thermal stress in livestock.

Alternatively, adjusting barn temperature and/or humidity has also been considered. For example, Barbari and Conti [18] studied the effect of cooling using an airstream or water over the floor on pregnant sows. Furthermore, the impact of a fogging system on the comfort of lactating sows [19] and fattening pigs [20] has also been studied.

Huynh et al. [21] stated that cooling systems, such as water baths, sprinklers, or an outdoor yard, showed positive effects on the physiologic responses, behavior, and productivity of pigs. Nevertheless, little information exists regarding the effects of such cooling systems applied in commercial conditions on carcass traits and pork quality parameters.

The objective of this study was to evaluate the effect of low-intensity showers provided at critical high-temperature daily hours on the body external temperature, feeding behavior and feeder occupancy ratio, carcass parameters, and meat quality characteristics of pigs.

## 2. Materials and Methods

All experimental procedures performed in this study complied with Spanish guidelines for the care and use of animals in research [22] and were completed under the protocols of the research committee of the Faculty of Veterinary Sciences of the Complutense University of Madrid (UCM).

All of the animals were housed in accordance with EU rules (European Directive 2008/120/EC). In compliance with European Directive 2010/63/EC Article 1, 5 (f), the present study did not involve any invasive procedure or treatment to the animals. For the animals, experimental design, and sample collection, the UCM certification is: O.H. (CEA)—UCM—NP0212072023-2023.

The study was carried out in commercial conditions on a feedlot located in Belinchón (Cuenca, Spain). For each experiment, 400 animals [200 barrows and 200 gilts, Large white × Landrace (PIC L65 × Camborough); RN- and halothane-negative] were randomly allotted in 40 pens (10 pigs/pen). Each pen was equipped with an electronic single-space feed dispenser, which consisted of a trough and a hopper connected through a load cell to a data logger.

A system of polyvinyl chloride pipes for water was installed over the pens on the surface of the ceiling. A shower nozzle was incorporated over 20 pens (10 with barrows and 10 with gilts), considering that the feed dispenser was not affected by the showers and that the distance from wall to wall was fully covered. Animals had free access to water. Further information related to feed composition can be found in Table S1. 

This experiment was carried out between June and September 2022, with an initial animal live weight of 66.5 ± 9.86 kg. Pigs received a low-intensity shower for 2 min in 30 min intervals from 12 h to 19 h, consistent with the warmest daily period (Figure S1) (15 showers/day; ~4 L/day of water per animal; SHO). A group without showers was also considered (CON).

At the end of the experimental period, the animals were sent to a commercial slaughterhouse (Incarlopsa, Tarancón, Cuenca, Spain), stunned with CO_2_, and slaughtered after a fasting period of 12 h (including fasting on farm, transport and lairage). Carcasses were eviscerated, split down the center of the vertebral column, weighed (HCW), and chilled at −1 °C (5 m/s; 90% relative humidity) for 150 min and stabilized for 20 h at −1 °C (0.5–1 m/s; 90% relative humidity). Samples of approximately 15 cm in size were taken from the *Longissimus Lumborum* (LL) muscle (at the level of the last rib).

### 2.1. Methods

#### 2.1.1. Feeder Occupancy Measurement

Feed access was recorded continuously in each experimental box by an electronic device located inside the feeder which registered each physical contact of the pig (MPIGDATA, Madrid, Spain). A one-minute interval was used to calculate feeder occupancy. Lack of physical contact was recorded as ‘free’, while any interaction was recorded as ‘occupied’. The hourly occupancy rate was calculated according to the proportion of minutes in one hour in which the feeder was considered occupied. 

#### 2.1.2. Thermographic Measures

A handheld infrared camera (Fluke Ti300, Fluke Ibérica S.L., Alcobendas, Spain), operated by trained personnel, was used to collect measures of the eyeball (EYE), the ear internal (EAR INT) and external (EAR EXT) points, the pig nose (NOSE), the pig shoulder (SHOULDER), the dorsal backfat at the level of the last rib (LOIN), the belly (BELLY), and the hip (HAM). Measures were collected 2 min before and 10 min after each shower every other day. A total of 3 animals per pen for each sex, therefore 120 animals (60 barrows and 60 gilts), were considered.

#### 2.1.3. Measurements at the Slaughterhouse: AutoFom III and pH

After bleeding and dehairing, the carcasses were scanned using AutoFom III technology (Frontmatec Smoerum A/S, Smørum, Denmark). Briefly, the pig carcass was scanned on the back as the hind legs were pulled on the conveyor and passed over the ultrasound transducer array. Scanning of the carcass generated an ultrasound image and 48 image parameters, thus providing information on skin, fat, and lean measures [23]. From those measurements, dorsal (LD) and gluteal (GL) fat thickness (mm), and lean meat yield (LMY, %) were estimated. 

A portable pH meter pH*K21 (NWK Binar, Puergen, Denmark) was used for pH measurement at 25 min, and 3, 20, and 40 h after slaughter in LL. Before pH measurement, the instrument was calibrated with pH 7.0 ± 0.02 and 4.0 ± 0.02 buffers and set to the corresponding temperature of measurement according to manufacturer specifications.

#### 2.1.4. Drip Loss

For the determination of weight loss during storage, an approximately 1 cm^3^ LL sample (weighing 10 ± 1 g) was placed under refrigerated conditions at 4 °C in a saturated atmosphere. Samples were weighed again after 72 h of storage. The difference between the final and initial weight was used to calculate the drip loss, which was expressed as a percentage of the initial weight [24].

#### 2.1.5. Color

Muscle LL color was evaluated on day 1 after slaughter using a Chroma Meter (CM 2002, Minolta, Camera, Osaka, Japan) calibrated against a white tile following the manufacturer’s recommendations [25]. The average of three random readings was used to measure lightness (L*), redness (a*), and yellowness (b*).

#### 2.1.6. Intramuscular Fat (IMF) Content and Fatty Acid (FA) Profile

Intramuscular fat (IMF) from LL was extracted and further methylated using the procedure described by Segura et al. [26]. Briefly, freeze-dried samples (50 ± 5 g frozen meat for 72 h at 25 °C and 0.2 bar; Lyoquest, Telstar, Tarrasa, Spain) were accurately weighed (200 ± 10 mg) in a safe-lock micro-test tube, homogenized in 1.5 mL dichloromethane/methanol (8:2 *v*/*v*), and mixed in a mixer mill (MM400, Retsch Technology, Haan, Germany). The final biphasic system was separated by centrifugation (8 min, 10,000 rpm) and the solvent was evaporated under a nitrogen stream. The previous extract was subjected to esterification by heating at 80 °C for 2 h in 1 mL of methanol/toluene/H_2_SO_4_ (88:10:2) and 1 mL of hexane. The fatty acid methyl esters (FAME) were separated using a gas chromatograph (HP 6890 Series GC System, Avondale, PA, USA) equipped with a flame ionization detector. The separation was performed with aHP-Innowax Polyethylene Glycol (30 m × 0.316 mm × 0.25 µm) JandW GC Column (Agilent Technologies GmbH, Waldbronn, Germany). Nitrogen was used as a carrier gas. After injection at 170 °C, the oven temperature was raised to 210 °C at a rate of 3.5 °C/min, then to 250 °C at a rate of 7 °C/min and held constant for 1 min. The flame ionization was held at 250 °C. FAME peaks were identified by comparing their retention times to those of authentic standards (Sigma-Aldrich, Alcobendas, Spain), and expressed as a percentage. Δ_9_ and Δ_5_-desaturase indexes were calculated according to Calvo et al. [27].

#### 2.1.7. Thiobarbituric Acid Reactive Substances (TBARS)

Oxidation status of meat (LL) was assessed by the thiobarbituric acid method [28]. A 27 mL measure of perchloric acid (3.83% *v*/*v*) was added to 5 g of meat and the mixture was homogenized with an Ultra-Turrax homogenizer (IKA, Barcelona, Spain) for 1 min and filtered. Aliquots were added to thiobarbituric acid (0.02 M) (1:1) and heated in boiling water for 25 min. A standard curve was prepared with 1,1,3,3-tetraethoxypropane, a precursor of malondialdehyde (MDA), at concentrations between 0 and 5 mg/L. Absorbance was measured at 532 nm and the values were expressed as mg MDA/kg meat.

### 2.2. Statistical Analysis

Data were analyzed following a completely randomized design using the general linear model (GLM) procedure contained in SAS [29]. Cooling treatment and sex were considered the fixed effects, and the pen was the random effect in the statistical model. Occupancy rate data were analyzed by chi-square procedure.

Data were presented as the mean of each group and the pooled standard deviation together with significance levels (*p*-value) of the main effects and interactions. Differences between means were considered statistically significant at *p* < 0.05.

## 3. Results

### 3.1. Body Temperature, Feeding Pattern, and Carcass Characteristics

**Figure 1:**
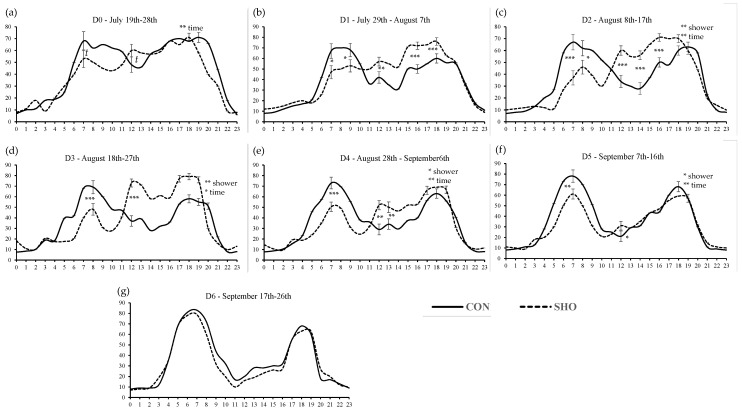
Feeding behavior (as % of feeder occupation) patterns with circadian variation for animals under low-intensity showers (SHO) and without (CON). * *p* < 0.05; ** *p* < 0.01; *** *p* < 0.0001.

The feeding behavior during different stages (D0–D6) is shown in Figure 1a–g and the temperature evolution in the feedlot is shown in Figure S1. A different pattern of behavior was observed between treatments. Overall, in CON, a shift in eating patterns to cooler periods of the day during elevated ambient temperatures was observed. A greater ingestion pattern was observed in the morning peak for CON animals than SHO. During the afternoon hours, which is the time of the highest HS, the opposite behavior was detected (Figure 1b–e). In period D5 (Figure 1f), minor differences were observed and, in D6 (Figure 1g), no differences between treatments were observed.

A temperature decrease after the shower was observed independently of the anatomical location (*p* < 0.005, Table 1). Overall, EAR INT, EAR EXT, and SHOULDER showed a difference higher than 2.0 °C, whereas a difference higher than 1.0 °C was observed for LOIN, NOSE, BELLY, and HAM. EYE measures showed the lowest decrease (0.5 °C). Except for EAR INT, a statistical interaction between sex and treatment was shown. Specifically, a higher decrease was detected for gilts (0.88, 3.87, 2.09, 2.73, 1.51, 1.97, and 1.43% for EYE, EAR EXT, NOSE, SHOULDER, LOIN, BELLY, and HAM, respectively) than for barrows (0.18, 1.16, 0.96, 1.50, 0.70, 0.78, and 0.82%, respectively; Table 1).

The treatment with showers (SHO) resulted in higher values of HCW, LMY, and fat thickness than the treatment without showers (CON). An increase of 4.72%, 3.87%, 11.8%, and 15.1% was detected for HCW, LMY, and fat thickness LD and GL, respectively (*p* < 0.01). In the case of fat thickness, such a difference was greater in gilts than in barrows (*p* < 0.05). Furthermore, gilts showed a 4.70% higher value of HCW (*p* < 0.0001) and a 2.39% higher value of LMY (*p* = 0.0885) than barrows (Table 2).

### 3.2. Meat Quality Parameters: pH, Water Holding Capacity, Color, Glycogen, Intramuscular Fat (IMF), Fatty Acid (FA) Profile, and Thiobarbituric Acid Reactive Substances (TBARS)

Pork from CON showed a 14.9% higher value of drip loss, and a 18.9% higher MDA concentration than SHO (*p* < 0.01) (Table 3). IMF content was 22.8% higher in SHO than in CON (*p* < 0.01). Furthermore, barrows showed higher IMF content than gilts (14.5%; *p* = 0.0744). Muscle glycogen levels were not affected by HS but a tendency to higher levels in gilts than in barrows was observed. Remarkably, higher L* (2.13%) and lower a* and b* (15.8% and 8.97%) values were observed for CON than SHO. No interaction S × T was detected.

**Figure 2:**
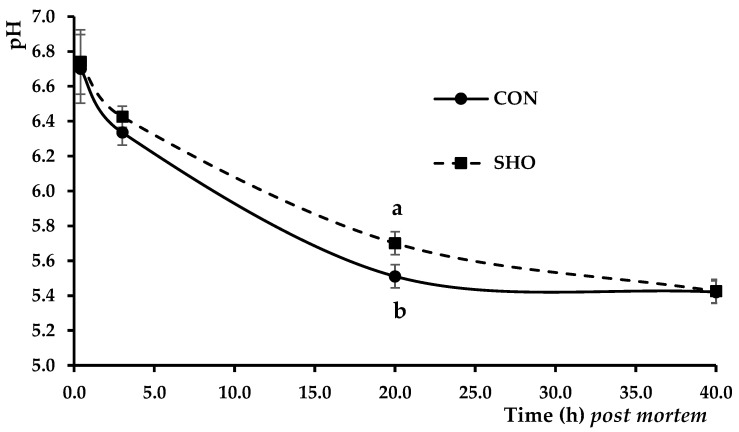
Evolution of LL pH values measured at 25 min, 3 h, 20 h, and 40 h *post mortem*. SHO = samples from shower treatment; CON = samples from control (non-showered) treatment. Different letters a, b, etc., indicate statistically significant differences (*p* < 0.05).

A decrease in pH values is shown in Figure 2. Interestingly, no differences between CON and SHO were observed at 25 min, 3 h, and 40 h. Nevertheless, CON pH_20h_ was significantly lower than SHO (*p* < 0.001), thus implying that a softer pH decrease happened in SHO LL.

Related to the subcutaneous fat outer layer (Table 4), CON C16:0 showed a 7.71% higher value than SHO and SHO C18:1n-9 showed a 5.71% higher value than CON (*p* < 0.05). Concurrently, CON ΣSFA showed a 7.15% higher value than SHO and SHO ΣMUFA and Δ_9_-desaturase indexes showed 6.08% (*p* < 0.001) and 5.81% (*p* < 0.05) higher values than CON.

Regarding the subcutaneous fat inner layer (Table 4), CON C18:0 and C18:2n-6 showed a 10.5% and 15.3% higher concentration than SHO, respectively. In addition, SHO C16:1n-7 and C18:1n-9 concentrations were 25.6% and 6.12% higher than CON. Concurrently, CON ΣSFA and ΣPUFA indexes were 5.42% and 12.9% higher than SHO. Furthermore, SHO ΣMUFA and Δ_9_-desaturase indexes showed 9.01% (*p* < 0.001) and 5.99% (*p* < 0.05) higher values than CON.

A higher amount of C18:1n-9 was detected in IMF from SHO than from CON pork (4.39% difference; *p* = 0.0085). Concurrently, higher ΣMUFA and Δ_9_-desaturase indexes were observed for SHO than CON (4.01% and 2.90% difference, respectively; *p* < 0.05) (Table 5). In addition, C18:2n-6 concentration showed a 12.3% higher value for CON than SHO (*p* < 0.01). Nevertheless, no statistically significant difference was detected for the ΣPUFA index.

## 4. Discussion

According to Poullet et al. [30], the upper limit of thermoneutrality, in which no extra energy is used for thermoregulation, ranges between 20 °C and 25 °C for growing–finishing pigs.

As the first parameter to be established, a different behavior towards feed intake (feeder attendance) was observed depending on CON and SHO. In addition, the showers implied differences in the body temperature of the animals.

An FI circadian rhythm has been described for growing–finishing pigs together with an age dependence. Although social rank and individual pig preferences or personalities should be considered, pigs show a fasting period at night but progress from consistent feeding throughout the day to two characteristic ingestion peaks (close to dawn and dusk, at lower ambient temperature) [31].

In line with our results, Cross et al. [32] described a shift in feeding patterns towards cooler periods of the day (late night or early morning) during the periods of higher ambient temperature, which was considered an adaptive mechanism to avoid consuming large amounts of food during the hottest part of the day to adapt to warmer environments by decreasing the amount of heat that needs to be dissipated into the environment. Reduced FI and increased body temperature have been widely described as related to HS. In terms of feeding behavior, the animals have more meals per day, but of shorter duration and in smaller quantities, a nibbling/slow eating pattern [4,5,30,33].

In agreement with our findings, the lower feeding rate observed in the HS group (CON) could be a cause of the lower body weight (BW) in HS compared to the thermoneutral (TN) group (SHO). Cervantes et al. [34] observed that FI level affected the postprandial body temperature of HS pigs, and its magnitude was larger after the afternoon and evening meals, thus suggesting a voluntary FI decrease during such periods. The interpretation of these results is complex, since they are influenced by many factors: daylight hours, the animal density (which increases as their BW increases), sex, etc. In other words, there are many factors to clarify and optimize. However, it can be concluded that the feeder occupancy rate was modified by low-intensity showers at precise hours.

To corroborate and support the results, higher body temperature for CON than for SHO was observed. In agreement with our results, Traulsen et al. [35] observed the highest correlation with ears, eyes, and the udder, and the lowest with vulva measurements, and established a quadratic relationship for all the locations except eyes and vulva. Also, Weschenfelder et al. [36] described ocular temperature as a good indicator of core temperature due to its proximity to the brain. Soerensen et al. [37] concluded that the higher the hair amount, the lower the accuracy when comparing skin emissivity and body temperature. Remarkably, most of the literature agrees that all the considered anatomical measurement points are suitable to establish qualitative changes in body temperature [38], thus corresponding with the results of this study.

The fact that heavier and fatter carcasses were obtained for the SHO than the CON group indicated higher FI in the SHO pigs than the CON ones. This confirms the observed data on the number of times the automatic feeder was accessed. In addition, the higher overall fat content of the SHO pigs resulted in an increase in IMF fat content, which significantly improved the meat quality of these pigs.

Regarding the literature, equivalent results were described by Dos Santos et al. [4] in growing pigs. The authors associated the higher values of HCW, lean content, and fat thickness of their TN than the HS group pigs with greater lipid and protein concentration increase. Lee et al. [11] carried out a meta-analysis and concluded that a linear inverse relationship can be stated between ADG, ADFI, and G/F of growing and finishing pigs and ambient temperature. Other authors described comparable results but using curvilinear models [39,40]. Srikanth et al. [41] described sexual dimorphism related to HS. Barrows have greater backfat thickness than gilts, consume more feed, and gain BW more rapidly. Correspondingly, gilts deposit proportionally more muscle and less fat in their carcass. This sex-specific difference in body composition could significantly alter the HS response, as it affects the animal’s ability to maintain its body temperature through radiant heat loss. Additionally, the effect of HS on immune cells was suggested to be dependent on an increase in the concentrations of prostaglandins, endogenous opioids, and glucocorticoids [41].

Noteworthily, our study was based on short showers at key times of the day, while most of the literature is based on heat relief strategies that are much more extensive in time and with higher resource costs. In this study, the results demonstrated that a punctual treatment, with minimum water consumption and applied at strategic times, can reverse most of the problems caused by HS in fattening pigs.

The pig housing conditions and/or production system can affect pork quality parameters, as the ambient temperature affects metabolic pathways for thermoregulation and FI, and consequently fat deposition and FA composition [42]. In our study, CON pork showed higher values of drip loss and MDA concentration than SHO, indicating an increase in oxidative stress in the CON group due to the environmental stress suffered by the pigs, thus showing the existence of a correlation with animal welfare.

Comparably to our results, Yang et al. [43], in pigs kept at 30 °C for 3 weeks, observed higher values of drip loss at 48 h, MDA content and L* at 24 h, and lower values of pH_24h_ and a* at 45 min, and b* at 24 h *post mortem* on HS than the TN group. Yang et al. and Gao et al. [43,44] concluded that a reduction in muscle carnosine content is one of the probable reasons for the negative impact of HS on meat quality and antioxidant capacity. Also, analogously to our results, Hao et al. [45] described lower pH_24h_ values on HS than the TN group. Although the authors did not observe variations in muscle glycogen levels, higher lactic acid concentration was described in muscles from HS pigs than from the TN group. The authors observed that hexokinase and pyruvate kinase activities, important for muscle glycolysis, were significant in the HS group. Permanent heat exposure for 21 d induced rapid AMPK (Adenine Monophosphate-Activated Protein Kinase) activation to phosphor-AMPK in muscle together with a positive correlation between AMPK activation to phosphor-AMPK and lactate accumulation, and negative with pH_24h_.

A well-known relationship between FA composition/profile and rheological properties [46], which directly affect the technological performance and odor and flavor components, is widely known [47].

Related to the C18:1n-9 concentration in this study, a direct relationship between ambient temperature and C18:0 desaturase activity was described a couple of decades ago [48,49]. In this line, over recent decades, improvements in the lipid composition of pork through dietary supplementation, gene editing, gut microbiota, etc., have been discovered in many studies, yet several key questions remain on the table. In this line, the environment is another factor affecting the lipid content. HS affects lipid metabolism by decreasing β-oxidation and lipolytic enzyme activities, thus leading to porcine adipose tissue-specific reactions and changes in FA content [50]. Specifically, Qu and Ajuwon found that HS increased de novo adipogenesis and TAG storage in porcine adipocytes through the phosphoenolpyruvate carboxykinase 1 (PCK1) pathway, thereby altering fat deposition and FA composition [51,52]. The SFA content in HS pigs was increased, and the MUFA and PUFA contents were decreased in these pigs.

Congruently, in agreement with our results, Qu and Ajuwon [51,52] observed, in serum and mesenteric fat tissue of HS pigs (35 °C, 7 d), an increase in C16:0 and C18:2n-6 concentrations. The C16:0 increase was associated with a potential role in the regulation of cellular membrane fluidity as part of the mechanism of homeoviscous adaptation to HS to maintain cellular function.

Zappaterra et al. [53] conducted a bibliographic revision of the non-genetic factors that describe the variability of IMF and FA profile of backfat, BF, and SM muscles. Lower IMF content on gilts slaughtered in summer than in winter was described. In addition, a dependence of the FA profile on the season was also observed, thus supporting the relationship of the FA profile with the membrane fluidity [53].

Heng et al. [54] indicated that HS improves the expression of lipid metabolism genes, including FA synthase, DGAT1, PPARg, SREBP1c, and FABP4, in the abdominal fat of pigs. Humans express two isoforms of stearoyl-CoA desaturase (SCD1 and SCD5), yet only SCD1 is thought to contribute to desaturation in lipogenic tissues [55]. Recent studies using knockout mouse models have revealed the phenotypes generated because of SCD1 gene deficiency. SCD1 is a component of the novel metabolic response of leptin signaling, upregulates insulin-signaling components, and affects glycogen metabolism [55].

Vessby et al. [56] described that insulin resistance is characterized by specific changes in FA composition in serum and skeletal muscle membranes. Impaired insulin sensitivity is associated with high concentrations of C16:0 and low levels of C18:2n-6 in serum, increased Δ_9_ and Δ_6_ activity, and decreased Δ_5_ desaturase activity. Ganesan et al. [57] observed that 12 h of HS blunted insulin signaling, decreased protein synthesis, and altered glycogen and FA metabolism. Seibert et al. [58], in pigs kept for 21 d at 38 °C, described higher MUFA and lower PUFA index values for TN abdominal fat, higher MUFA, and lower SFA for the TN inner layer of subcutaneous fat and higher MUFA and lower SFA and PUFA for the TN outer layer of subcutaneous fat. The authors observed an increase in circulating insulin in HS and hypothesized an upregulation of desaturase abundance and activity, promoting FA desaturation, but coexisting with the need for membrane fluidity [58].

The literature regarding the effect of alleviating HS using low-intensity showers on pork quality is scarce. The improvement of this strategy by optimizing the shower schedule and duration remains to be studied together with the consideration of diet and genetics.

## 5. Conclusions

Animals subjected to short showers at critical times showed lower body temperature and higher feeder occupancy rate, body weight, lean meat yield, and fattening (including IMF) than those not showered, thus increasing carcass quality. In addition, fatty acid profiles showed a dependence on heat stress, where the use of showers led to an increase in MUFA and a decrease in SFA and PUFA fatty acids, thus implying a different activity of the desaturase enzymes with heat. 

Accepting that heat stress must be considered as another variable to be optimized in animal production, the alleviation of heat stress with showers at critical times should be considered as an interesting tool that improves on the one hand, carcass and meat quality, and, on the other, animal welfare of the pigs.

## Figures and Tables

**Table 1:** Effect of low-intensity showers (SHO) provided to fattening gilts and barrows on external surface temperature measured in different anatomical regions.

Anatomical Location	Gilts (*n =* 60)				Barrows (*n =* 60)				RMSE ^1^	*p*-Value ^2^		
	CON		SHO		CON		SHO			S	T	S × T
Eye	36.74	^a^	35.86	^b^	36.27	^ab^	36.09	^b^	0.1974	NS	**	*
Ear INT	36.83	^a^	34.67	^b^	36.42	^a^	34.41	^b^	0.3430	NS	***	NS
Ear EXT	37.29	^a^	33.42	^c^	36.97	^a^	35.81	^b^	0.6084	*	***	**
Nose	33.76	^a^	31.67	^b^	33.52	^a^	32.57	^ab^	0.2894	NS	**	0.0524
Shoulder	37.68	^a^	34.95	^c^	37.33	^a^	35.83	^b^	0.5289	NS	***	*
Loin	37.40	^a^	35.89	^c^	37.41	^a^	36.71	^b^	0.4003	*	***	*
Belly	37.85	^a^	35.88	^b^	37.79	^a^	37.01	^a^	0.3953	0.0685	***	*
Ham	37.39	^a^	35.96	^c^	37.43	^a^	36.61	^b^	0.4198	0.0894	***	0.0828

^1^ RMSE = root mean square error (120 animals, 2 sexes, and 2 treatments). ^2^ T = treatment (showers (SHO) vs. control (CON)); S = sex (gilts vs. barrows). *p*-value: NS for *p* > 0.05; * *p* < 0.05; ** *p* < 0.01; *** *p* < 0.0001. Different letters a, b… within a row indicate statistically significant differences (*p* < 0.05).

**Table 2:** Effect of low-intensity showers (SHO) provided to fattening gilts and barrows on hot carcass weight (HCW), lean meat yield (LMY), and fat depth over the muscle *Longissimus dorsi* (LD) at the level of the last rib, and over the muscle *Gluteus superficialis* (GL).

		CON (*n =* 192)				SHO (*n =* 190)				RMSE	*p*-Value		
		♀		♂		♀		♂			S	T	S × T
HCW (kg)		92.98	^b^	88.95	^c^	97.95	^a^	93.00	^b^	1.099	***	***	NS
LMY (%)		58.99	^b^	57.39	^c^	61.17	^a^	59.90	^ab^	1.548	0.0885	*	NS
Fat width (mm)	LD	16.51	^b^	17.91	^ab^	19.05	^a^	19.20	^a^	1.298	NS	**	*
	GL	11.88	^b^	12.88	^ab^	14.01	^a^	14.32	^a^	1.377	NS	*	*

RMSE = root mean square error. T = treatment (showers (SHO) vs. control (CON)); S = sex (gilts (♀) vs. barrows (♂)). *p*-value: NS for *p* > 0.05; * *p* < 0.05; ** *p* < 0.01; *** *p* < 0.0001. Different letters a, b… within a row indicate statistically significant differences (*p* < 0.05).

**Table 3:** Effect of low-intensity showers (SHO) provided to fattening gilts and barrows on meat quality characteristics at carcass cut-out time.

	CON (*n =* 20)				SHO (*n =* 20)				RMSE	*p*-Value		
	♀		♂		♀		♂			S	T	S × T
Drip loss (%)	12.08	^a^	12.56	^a^	10.48	^b^	10.49	^b^	1.033	NS	**	NS
TBARS (mg MDA/kg)	0.382	^a^	0.421	^a^	0.321	^b^	0.330	^b^	0.0301	NS	**	NS
IMF (%)	2.041	^b^	2.557	^ab^	2.822	^a^	3.131	^a^	0.517	0.0744	**	NS
Glycogen (µmol/g)	1.000	^a^	0.800	^b^	1.095	^a^	0.865	^b^	0.2000	0.0712	NS	NS
L*	56.19	^b^	57.67	^a^	55.38	^c^	56.06	^b^	1.281	*	*	NS
a*	3.712	^b^	3.892	^b^	4.297	^a^	4.738	^a^	0.4193	NS	*	NS
b*	6.584	^b^	6.842	^ab^	7.060	^ab^	7.689	^a^	0.7403	0.0550	*	NS

RMSE = root mean square error (40 samples: 2 sexes and 2 treatments, *n =* 10); TBARS = thiobarbituric reactive substances; MDA = malondialdehyde; IMF = intramuscular fat. T = treatment (showers (SHO) vs. control (CON)); S = sex (gilts (♀) vs. barrows (♂)). *p*-value: NS for *p* > 0.05; * *p* < 0.05; ** *p* < 0.01. Different letters a, b… within a row indicate statistically significant differences (*p* < 0.05).

**Table 4:** Effect of low-intensity showers (SHO) provided to fattening gilts and barrows on fatty acid composition (%) and estimation of delta-9 (Δ_9_) and delta-5 (Δ_5_) desaturase activity in outer and inner backfat layers of subcutaneous fat.

		CON (*n =* 20)				SHO (*n =* 20)				RMSE	*p*-Value		
		♀		♂		♀		♂			S	T	S × T
INNER													
	C16:0	22.61		22.74		22.17		22.09		1.013	NS	NS	NS
	C16:1n-7	1.506	^d^	1.840	^c^	2.034	^b^	2.464	^a^	0.2693	**	**	NS
	C18:0	14.70	^a^	14.53	^a^	13.10	^b^	13.05	^b^	0.5885	NS	*	NS
	C18:1n-9	38.52	^b^	37.60	^c^	40.71	^a^	40.37	^a^	0.8237	0.1311	*	0.0999
	C18:2n-6	15.81	^a^	15.91	^a^	13.60	^b^	13.25	^b^	0.8206	NS	**	NS
	ΣSFA	39.16	^a^	39.21	^a^	37.10	^b^	37.02	^b^	0.9290	NS	*	NS
	ΣMUFA	43.71	^b^	43.69	^b^	47.75	^a^	48.31	^a^	1.685	NS	**	NS
	ΣPUFA	17.13	^a^	17.09	^a^	15.15	^b^	14.67	^b^	1.555	NS	**	NS
	Δ_9_	0.5090	^b^	0.5051	^b^	0.5388	^a^	0.5400	^a^	0.0202	NS	*	NS
	Δ_5_	0.7042	^b^	0.7812	^a^	0.6968	^b^	0.7885	^a^	0.0302	*	NS	NS
OUTER													
	C16:0	22.77	^a^	22.67	^a^	21.01	^b^	20.93	^b^	0.9656	NS	*	NS
	C16:1n-7	2.292	^b^	2.671	^ab^	2.444	^ab^	2.958	^a^	0.2582	*	NS	NS
	C18:0	11.54		11.56		10.90		10.73		0.9881	NS	NS	NS
	C18:1n-9	39.91	^b^	38.57	^b^	41.90	^a^	41.33	^a^	1.184	NS	*	NS
	C18:2n-6	15.88		15.93		15.45		15.41		1.270	NS	NS	NS
	ΣSFA	36.35	^a^	36.24	^a^	33.84	^b^	33.55	^b^	1.783	NS	**	NS
	ΣMUFA	46.09	^b^	46.20	^b^	48.90	^a^	49.36	^a^	1.401	NS	**	NS
	ΣPUFA	17.57		17.57		17.25		17.09		1.393	NS	NS	NS
	Δ_9_	0.5414	^b^	0.5365	^b^	0.5713	^a^	0.5731	^a^	0.0182	NS	*	NS
	Δ_5_	0.6985	^c^	0.7712	^b^	0.7382	^c^	0.8029	^a^	0.0272	*	*	NS

∑SFA = sum of saturated fatty acids (C14:0 + C15:0 + C16:0 + C17:0 + C18:0 + C20:0), ∑MUFA = sum of monounsaturated fatty acids (C14:1n-5 + C16:1n-7 + C16:1n-9 + C17:1 + C18:1n-7 + C18:1n-9 + C20:1n-9), ∑PUFA = sum of polyunsaturated fatty acids (C18:2n-6 + C18:3n-6 + C18:3n-3 + C18:4n-3 + C20:3n-6 + C20:4n-6 + C20:5n-3). Δ_9_ and Δ_5_-desaturase indexes calculated according to [27]. RMSE = root mean square error (40 samples; 2 sexes and 2 treatments: *n =* 10). T = treatment (showers (SHO) vs. control (CON)); S = sex (gilts (♀) vs. barrows (♂)). *p*-value: NS for *p* > 0.05; * *p* < 0.05; ** *p* < 0.01 Different letters a, b… within a row indicate statistically significant differences (*p* < 0.05).

**Table 5:** Effect of low-intensity showers (SHO) provided to fattening gilts and barrows on fatty acid composition (%) and estimation of delta-9 (Δ_9_) and delta-5 (Δ_5_) desaturase activity in fatty acids of intramuscular fat of LL.

	CON (*n =* 20)				SHO (*n =* 20)				RMSE	*p*-Value		
	♀		♂		♀		♂			S	T	S × T
C16:0	23.44	^a^	22.36	^ab^	23.14	^a^	21.87	^b^	1.217	***	0.1213	NS
C16:1n-7	3.203	^a^	3.064	^ab^	3.422	^a^	2.858	^b^	0.4340	*	NS	0.1451
C18:0	12.80	^a^	12.07	^ab^	11.83	^b^	12.25	^ab^	0.8646	NS	NS	0.0782
C18:1n-9	38.44	^b^	37.40	^c^	40.58	^a^	38.74	^b^	2.004	*	*	NS
C18:2n-6	11.47	^ab^	13.12	^a^	9.921	^b^	11.63	^ab^	1.858	*	*	NS
ΣSFA	38.07	^a^	36.24	^b^	36.82	^ab^	35.91	^b^	1.728	*	0.1650	0.1898
ΣMUFA	45.98	^bc^	45.21	^c^	48.69	^a^	46.31	^bc^	2.351	*	*	0.0551
ΣPUFA	15.95	^ab^	18.56	^a^	14.49	^b^	17.78	^a^	3.235	**	0.1405	0.0662
Δ_9_	0.5256	^c^	0.5314	^bc^	0.5476	^a^	0.5409	^ab^	0.0147	NS	*	NS
Δ_5_	0.8700		0.8753		0.8758		0.8775		0.0157	NS	NS	NS

∑SFA = sum of saturated fatty acids (C14:0 + C16:0 + C17:0 + C18:0 + C20:0), ∑MUFA = sum of monounsaturated fatty acids (C14:1n-5 + C16:1n-7 + C16:1n-9 + C17:1 + C18:1n-7 + C18:1n-9 + C20:1n-9 + C22:1n-9), ∑PUFA = sum of polyunsaturated fatty acids (C18:2n-6 + C18:3n-6 + C18:3n-3 + C18:4n-3 + C20:3n-6 + C20:4n-6 + C20:5n-3 + C22:4n-6 + C22:5n-3 + C22:6n-3). Δ_9_ and Δ_5_ desaturase indexes calculated according to [27]. RMSE = root mean square error (40 samples; 2 sexes and 2 treatments: *n =* 10). T = treatment (showers (SHO) vs. control (CON)); S = sex (gilts (♀) vs. barrows (♂)). *p*-value: NS for *p* > 0.05; * *p* < 0.05; ** *p* < 0.01; *** *p* < 0.0001. Different letters a, b… within a row indicate statistically significant differences (*p* < 0.05).

## Data Availability

Data available on request due to restrictions.

## Supplementary Materials

The following supporting information can be downloaded at: https://www.mdpi.com/article/10.3390/ani14111661/s1, Table S1: Ingredient composition and nutrient content of the diet. Figure S1: Mean hourly temperature values for each of the periods considered.
