# Supplementary material for: Alleviating Heat Stress in Fattening Pigs: Low-Intensity Showers in Critical Hours Alter Body External Temperature, Feeding Pattern, Carcass Composition, and Meat Quality Characteristics
Source: Animals (Basel). 2024 Jun 1;14(11):1661. doi: 10.3390/ani14111661 (PMC11171127; doi:10.3390/ani14111661)
Supplement: Supplementary file 1 [file animals-14-01661-s001.zip › animals-3012841-supplementary.pdf]

Table S1. Diet ingredients.

**Ingredients (%)**	
Barley	45.80
Corn	35.00
Soybean meal 47%	13.80
Fat 3/5	2.180
Sugar cane molasses	1.000
Dicalcium Phosphate	0.397
Sodium Chloride	0.444
Calcium Carbonate	0.997
L-Lysine	0.202
DL-Methionine	0.023
L-Threonine	0.040
Premix	0.200
**Chemical composition**	
Moisture (%)	11.0
EN (kcal/kg)	2400
Ash (%)	4.29
Crude Protein, (PB)%	14.0
Crude Fat (%)	4.34
Linoleic acid (%)	1.14
FND (%)	13.3
FAD (%)	4.52
Starch (%)	46.7
Lysine DIS (%)	0.70
Methionine DIS (%)	0.22
Met + Cys DIS (%)	0.43
Threonine DIS (%)	0.46
Tryptophan DIS (%)	0.13
Calcium (%)	0.60
Phosphorus dig (%)	0.23
Sodium (%)	0.18
Chlorine (%)	0.38

Figure S1. Mean hourly temperature values for each of the periods considered.

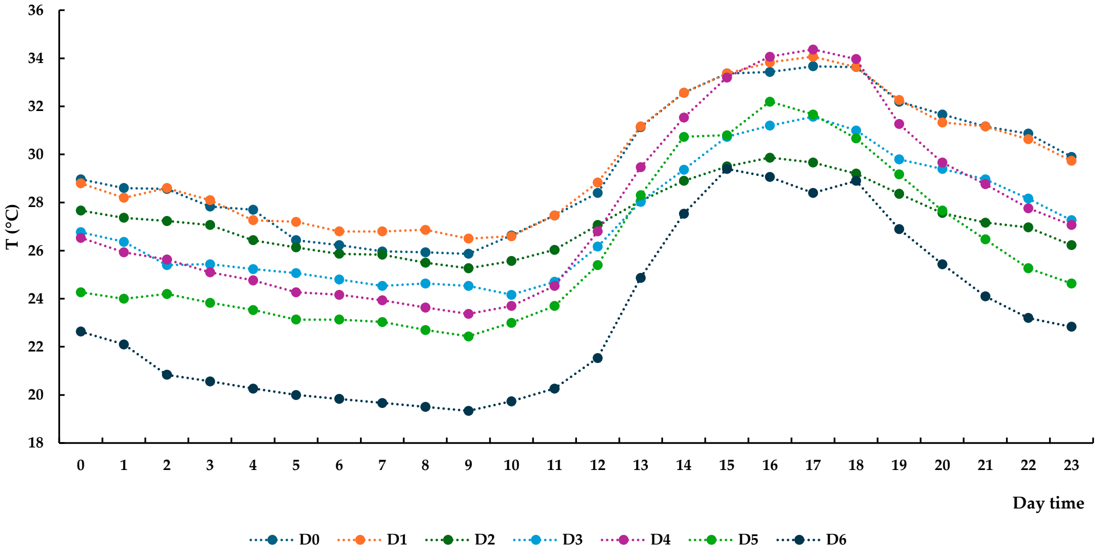

D0, D1, etc. according to Figure 1.
